# Chlorogenic Acid Attenuates Kidney Ischemic/Reperfusion Injury via Reducing Inflammation, Tubular Injury, and Myofibroblast Formation

**DOI:** 10.1155/2019/5423703

**Published:** 2019-09-22

**Authors:** Nur Arfian, Danny A. P. Wahyudi, Ingesti B. Zulfatina, Arsitya N. Citta, Nungki Anggorowati, Ali Multazam, Muhammad M. Romi, Dwi C. R. Sari

**Affiliations:** ^1^Department of Anatomy, Faculty of Medicine, Public Health and Nursing, Universitas Gadjah Mada, Yogyakarta, Indonesia; ^2^School of Medicine, Faculty of Medicine, Public Health and Nursing, Universitas Gadjah Mada, Yogyakarta, Indonesia; ^3^Department of Anatomical Pathology, Faculty of Medicine, Public Health and Nursing, Universitas Gadjah Mada, Yogyakarta, Indonesia; ^4^Master Program in Biomedical Science, Faculty of Medicine, Public Health and Nursing, Universitas Gadjah Mada, Yogyakarta, Indonesia; ^5^Department of Physiotherapy, Universitas Muhammadiyah Malang, Malang, East Java, Indonesia

## Abstract

Kidney ischemic/reperfusion (I/R) injury is the main cause of acute kidney injury (AKI) involving renal function deterioration, renal architecture damage, and inflammation. This condition may lead to kidney fibrosis with epithelial to mesenchymal transition (EMT) and myofibroblast formation. Inhibition of chronic effects of kidney I/R injury may provide effective strategies for treating AKI and chronic kidney diseases (CKDs). Chlorogenic acid (CGA) is recognized as a powerful antioxidant, with anti-inflammatory and antifibrotic properties in many conditions. However, the effect of CGA on kidney I/R injury has not been elucidated yet. Kidney I/R injury was performed on male Swiss background mice (I/R group, *n* = 5, 3-4 months, 30–40 g) which underwent bilateral renal pedicles clamping for 30 minutes and then were euthanized on day three after operation. Three groups of I/R were treated with 3 different doses of CGA intraperitoneally for 2 days: 3.5 (I/R + CGA1 group), 7 (I/R + CGA2 group), and 14 (I/R + CGA3 group) mg/kg of body weight. Tubular injury was quantified based on Periodic Acid-Schiff staining, while reverse transcriptase PCR (RT-PCR) was performed to quantify mRNA expression of TGF-*β*1, vimentin, SOD-1, TLR-4, TNF-α, NF-κB and MCP-1. Immunohistochemical staining was done to quantify proliferating cell nuclear antigen (PCNA), myofibroblast (*α*-SMA), SOD-1 and macrophage (CD68) number. Kidney I/R demonstrated tubular injury and increased inflammatory mediator expression, macrophage number, and myofibroblast expansion. Meanwhile, histological analysis showed lower tubular injury with higher epithelial cell proliferation in CGA-treated groups compared to the I/R group. RT-PCR also revealed significantly lower TGF-*β*1 and vimentin mRNA expressions with higher SOD-1 mRNA expression. CGA-treated groups also demonstrated a significantly lower macrophage and myofibroblast number compared to the I/R group. These findings associated with lower mRNA expression of TLR-4, TNF-α, NF-κB, and MCP-1 as inflammatory mediators in CGA groups. I/R + CGA3 represented the highest amelioration effect among other CGA-treated groups. CGA treatment attenuates kidney I/R injury through reducing inflammation, decreasing myofibroblast expansion, and inducing epithelial cells proliferation.

## 1. Introduction

Acute kidney injury (AKI) incidence has increased in the last two decades. AKI incidence reached 550 per 100,000 in population in 2003 [1]. In hospitals, the incidence ranges from 2% to 7%, and 5 to 10% in intensive care units (ICU) [2]. Epidemiological data identified different causes of AKI between developed and developing countries. In the developed countries, most AKI patients are elderly, while, predominantly, patients with AKI in developing countries are younger populations with prerenal conditions as the main cause [3].

Kidney ischemic/reperfusion (I/R) injury is the main cause of AKI. This condition has high morbidity and mortality, involving high costs for the treatment. Kidney I/R injury is also seen as a risk factor for chronic kidney disease (CKD) [4]. Kidney I/R injury, characterized by reduction and loss of renal blood flow (stenosis), is followed by reperfusion. Hypoxia induces epithelial cell injury and apoptosis, which are the main consequence of hypoxia [5]. Hypoxia causes cell injury and dysfunction with failure of the Na^+^-K^+^ ATPase pump and increases intracellular calcium ions, thus producing cell death and oxidative stress [6]. Epithelial cell proliferation and regeneration occur after injury [7]. Tubular epithelial cells undergo epithelial to mesenchymal transition (EMT) or dedifferentiation. The surviving epithelial cells will replace apoptotic cells after AKI [8]. However, it is known that AKI with kidney I/R injury might lead to CKDs. In most cases of AKI, EMT type II after kidney I/R injury plays an important role in renal fibrogenesis with incomplete regeneration and leads to interstitial fibrosis [9]. Furthermore, reperfusion itself exaggerates reactive oxygen species (ROS) production, causing more severe injury after hypoxia [10].

I/R injury also exaggerates metabolic imbalance that leads to inflammation [11]. During I/R injury, the kidneys produce Toll-like receptors-2 and -4 (TLR-2 and TLR-4), thus inducing sterile inflammation. TLRs activation produces upregulation of proinflammatory mediators such as TNF-*α*, MCP-1, IL-8, IL-6, IL-1*β*, and TGF-*β*. Cytokines and chemokines induce infiltration of macrophages [5]. TGF-*β* plays a role in the fibrotic cascade and induces fibroblast proliferation, inhibits collagen deposition, and stimulates collagen production, fibronectin, and proteoglycan [12]. Fibroblast activation changes the cell phenotype to become myofibroblasts with *α*-SMA marker expression [13]. TGF-*β* is also important for protecting epithelial cells from sublethal injury [14]; however, overstimulation to fibroblasts and epithelial cells produces accumulation of an extracellular matrix and leads to interstitial fibrosis [15]. In chronic CKD conditions, kidney fibrosis produces progressive and irreversible renal function deterioration [12].

Chlorogenic acid (CGA) is a polyphenol substance containing caffeic and quinic acid [16] which are found in many vegetable products [17]. It is also found in green coffee. It has many positive effects, and one of them is reducing I/R injury in many organs with its anti-inflammation effects such as inhibiting TNF-*α* and IL-6 activation and reducing NF-*κ*B expression [18, 19]. CGA also plays an important role as an antioxidant substance through eliminating free radicals [20, 21] and promoting antifibrotic [22] effects. CGA treatment attenuated hepatic ischemic/reperfusion injury in rat due to its antioxidant effects [23]. However, the effects of CGA on kidney I/R injury have not been elucidated yet. This study aimed to elucidate the effects of CGA on kidney I/R injury and demonstrate the renoprotective effects of CGA on kidney I/R injury, especially in tubular injury, inflammation, and fibrosis mechanism.

## 2. Materials and Methods

### 2.1. Animal Subjects and Kidney Ischemic/Reperfusion (IR) Injury Model

Swiss background mice (male, *n* = 25, 3-4 months, 20–30 grams body weight) were used for this study. Mice were maintained according to the standard laboratory conditions with the provision of diet and water ad libitum and 12-hour light and dark cycle cages. This study had been approved by the Ethical Committee of the Faculty of Medicine, Public Health and Nursing, Universitas Gadjah Mada, with the reference number of KE/FK/1066/EC/2017. Bilateral renal pedicles clamping was performed to induced kidney IR injury based on previously published methods [24]. Briefly, the mice were anesthetized with sodium pentobarbital 0.1 ml/10 g body weight. After that, the abdomen was opened. Next, both renal pedicles were visualized, clamped with a nontraumatic vascular clamp for 30 minutes, and then removed. Sham operation (SO group, *n* = 5) procedure was used for the control group with only opening the mice's abdomen without renal pedicles clamping. Chlorogenic acid (CGA) (Sigma Aldrich, C3878) was administered through intraperitoneal injection daily, based on the previous results in rat [23]. We converted the dose according to the conversion factor of rat (200 gr/0.2 kg) to mouse (20 gr/0.02 kg) based on the body weight, which was 0.14 [25]. Then, the formula used for dose quantification was (dose in rat (mg/kg) × BW of rat (0.2 kg) × conversion factor (0.14))/BW of mouse (0.02 kg). The doses of the rat were 2.5, 5, and 10 mg/kg BW, which were equal to 3.5, 7, and 14 mg/kg BW based on the formula. Mice were divided into the following groups: SO group with NaCl 0.9% intraperitoneal injection; I/R group (*n* = 5); I/R group with CGA dose 3.5 mg (I/R + CGA1 group, *n* = 5), dose 7 mg (I/R + CGA2 group, *n* = 5), and dose 14 mg (I/R + CGA3 group, *n* = 5)/kg of body weight (BW). On the following day after the operation, the mice were injected with CGA. Mice were euthanized at day 3 after the operation.

### 2.2. Euthanizing and Kidney Harvesting

Mice were anesthetized with intraperitoneal injection of sodium pentobarbital (0.1 ml/10 g body weight). After opening the abdomen and thorax, perfusion of the organ was done via the left ventricle using 0.9% NaCl. Kidneys were harvested, and then, the right kidney was placed into RNA preservation solution for RNA extraction and the left kidney was fixated in normal buffer formalin (NBF) solution for 24 hours and then used for paraffin making.

### 2.3. Histological Analysis and Immunohistostaining

Paraffin-embedded sections with 4 *μ*m thickness were used for basic staining and immunohistochemical (IHC) staining. Paraffin sections were deparaffinized and rehydrated using serial solutions of xylene and alcohol and then stained with Periodic Acid-Schiff (PAS) to determine and quantify the tubular injury score. For IHC staining, deparaffinized and rehydrated steps were followed by antigen retrieval using heating in citrate buffer pH 6, blocking peroxidase using H_2_O_2_ 3% in PBS solution, and then blocking nonspecific antigen using background sniper (Star Trek IHC kit, BioCare Medical). Afterwards, the slides were incubated with *α*-smooth muscle actin (*α*-SMA) (1 : 400, Sigma, A2547), CD68 (1 : 400, Abcam, ab125212), proliferating cell nuclear antigen (PCNA) (1 : 200, Abcam, ab29), and super oxide dismutase-1 (SOD-1) (1 : 100, Bioss, bs-10216R) as the 1st antibodies for overnight incubation in 4°C and then provided with appropriate 2nd antibody Trekkie Universal Link, TrekAvidin-HRP, and diaminobenzidine-tetrahydrochloride (DAB) (Biocare Medical®). The *α*-SMA antibody immunostaining was used for measuring myofibroblast expansion, CD68 antibody for counting macrophage, and proliferating cell nuclear antigen (PCNA) for assessing epithelial cell proliferation in the sample kidneys. Later, the quantification was measured by counting the positive staining signal from 15 fields for each sample with 400x magnification using ImageJ software. SOD-1 immunostaining was used to localize SOD-1 expression in the tissue.

### 2.4. Tubular Injury Score

Tubular injury score was assessed by PAS staining and was determined using a semiquantitative scoring system in 15 fields for each specimen with 200x magnifications. The variables of scoring were as follows: (1) tubular atrophy and dilatation, (2) loss of brush border, (3) accumulation of inflammatory cells, and (4) intraluminal cast. Otherwise, the scale of injury was from 0 to 4: score 0: normal condition; score 1: mild (injury <25%); score 2: moderate (injury 25–50%); score 3: severe (injury 50–75%); and score 4: extensive damage (injury >75%).

### 2.5. Reverse Transcriptase PCR (RT-PCR)

RNA was extracted using Genezol solution (Genezol, Cat. no. GZR100), followed by the RNA concentration quantification using nanodrop ReverTra Ace® (Toyobo, Japan, Cat. no. TRT-101), and random primers (Toyobo, Japan, Cat. no. 3801) were used in synthesizing the cDNA and then conducting reverse transcriptase PCR (RT-PCR) using Master Mix (Go Taq green) for assessing the expression of the following genes: (1) Toll-like receptor-4/TLR-4 (forward 5′-GGGCCTAAACCCAGTCTGTTTG-3′, reverse 3′-GCCCGGTAAGGTCCATGCTA-5′); (2) monocyte chemoattractant protein-1/MCP-1 (forward 5′-GGCATCACAGTCCGAGTCACAC-3′, reverse 3′-CTACAGACAACC-ACCTCAAGCACTTCTGTAG-5′); (3) vimentin (forward 5′-CGGAAAGTGGAATCCTTGCA-3′, reverse 5′-CACATCGATCTGGACATGCTG-3′); (4) tumor necrosis factor-*α*/TNF-*α* (forward 5′-GCCTCTTCTCATTCCTGCTTG-3′, reverse 3′-CTGATGAGAGGGAGGCCATT-5′); (5) nuclear factor-*κ*B/NF-*κ*B (forward 5′-GCGTACACATTCTGGGGAGT-3′, reverse 3′-ACCGAAGCAGGAGCTATCAA-5′); (6) super oxide dismutase-1/SOD-1 (forward 5′-AGCATTCCATCATTGGCCGTA-3′, reverse 3′-TTTCCACCTTTGCCCAAGTCA-5′); and (7) GAPDH (forward 5′-TTGCTGTTGAAGTCGCAGGAG-3′, reverse 3′-TGTGTCCGTCGTGGATCTGA-5′) were used as reference. The gene expressions were quantified using densitometry analysis (ImageJ software), and the *GAPDH* gene was used to normalize the gene expressions (house-keeping gene).

### 2.6. Statistical Analysis

Statistical analysis was done using SPSS 22 for Windows (IBM, Chicago). The distribution of the data was priorly determined using the Shapiro–Wilk test and presented as mean ± standard error measurement (SEM) to be analyzed by parametric tests using one-way ANOVA. For multiple comparisons, the data were then examined using the post hoc LSD test. The level of statistical significance was *p* < 0.05.

## 3. Results

### 3.1. CGA Treatment Attenuated Kidney Injury and Tubular Injury and Increased Epithelial Cell Proliferation after Kidney I/R Injury

**Figure 1:**
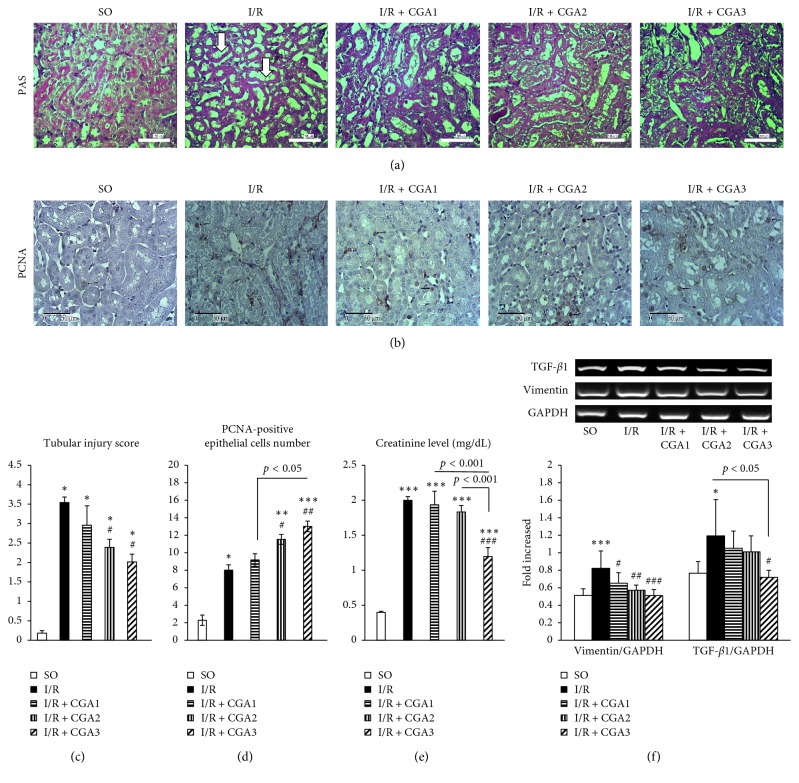
(a) Representative pictures of PAS staining in all groups. The SO group had normal histology of kidney tissue with brush border. The IR group had tubular injury characteristics with dilatation and brush-border loss (white arrow). (b) Immunostaining of PCNA showed positive staining in epithelial cells' nuclei (black arrows). (c, d). Quantification of tubular injury score and PCNA-positive cell number showed attenuation of tubular injury which associated with the increased number of proliferating epithelial cells in CGA-treated groups (especially IR + CGA3 group). (e) Creatinine level measurement showed reduction in the IR + CGA3 group. (f) Reverse transcriptase-PCR (RT-PCR) analysis of vimentin (mesenchymal marker) and TGF-*β*1 (profibrotic factor). The IR + CGA3 group demonstrated significantly lower vimentin and TGF-*β*1 mRNA expressions with the IR group. ^*∗*^*p* < 0.05 vs. SO group; ^*∗∗*^*p* < 0.01 vs. IR group; ^*∗∗∗*^*p* < 0.001 vs. SO group. ^#^*p* < 0.05 vs. IR group; ^##^*p* < 0.01 vs. IR group; ^###^*p* < 0.001 vs. IR group.

Kidney I/R injury demonstrated kidney injury with higher tubular injury and creatinine level. Damaged renal architecture in the IR group was demonstrated with tubular cell apoptosis, tubular dilatation and atrophy, brush border loss, intraluminal cast formation, and macrophage infiltration (Figure 1). Quantification of tubular injury showed higher scores in the I/R group compared to the SO group.

Amelioration of renal architecture was shown in the CGA group, which was demonstrated by significantly lower tubular injury scores in I/R CGA2 and I/R + CGA3 groups compared to the I/R group. CGA treatment also induced proliferation of epithelial cells. Quantification of PCNA IHC staining as a key proliferation marker revealed significantly higher proliferation of epithelial cells in the I/R group compared to SO. Furthermore, I/R + CGA2 and I/R + CGA3 groups had higher proliferation of epithelial cell number compared to the I/R group. I/R + CGA3 had the highest number of proliferating epithelial cells. These findings associated with amelioration of kidney function in CGA-treated groups, as shown with significantly lower serum creatinine levels in the IR + CGA3 group compared to the IR group. The IR group also demonstrated higher mesenchymal marker, vimentin and profibrotic marker, and TGF-*β*1. Meanwhile, CGA-treated groups represented lower vimentin and TGF-*β*1 mRNA expressions compared to the I/R group. IR + CGA3 had significantly lower vimentin and TGF-*β*1 mRNA expressions compared to the IR group.

### 3.2. CGA Attenuated Myofibroblast Expansion and Upregulated SOD-1 Expression

**Figure 2:**
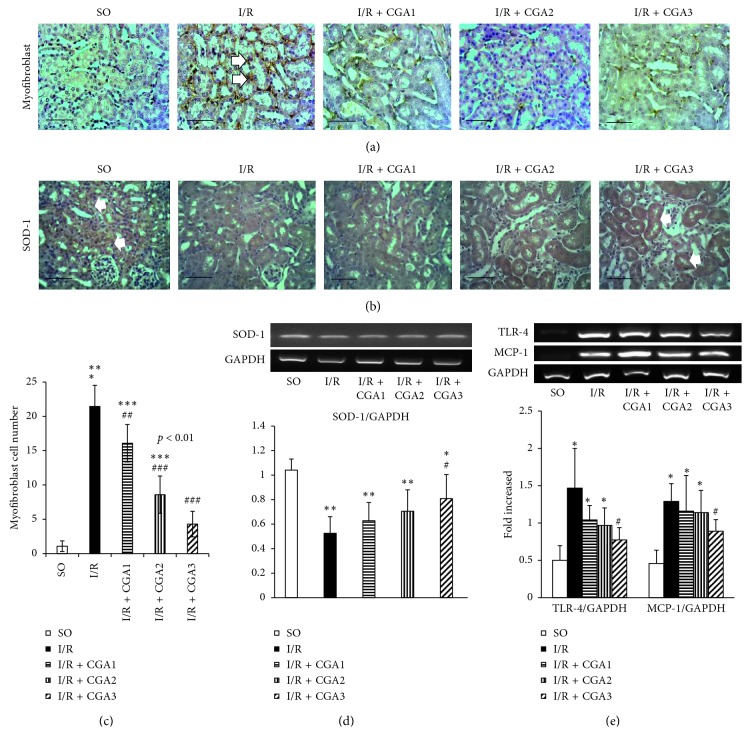
(a) Representative pictures of *α*-SMA immunostaining for myofibroblast marker. Positive staining is shown in interstitial areas (white arrow). (b) Representative pictures of SOD-1 immunostaining. Positive staining is shown in epithelial cells in SO, meanwhile reduction in the IR group. The IR + CGA3 group demonstrated positive staining in epithelial cells. (c) Quantification of the myofibroblast number showed reduction of the numbers in CGA-treated groups, especially the IR + CGA3 group. (d) Reverse transcriptase-PCR (RT-PCR) analysis of SOD-1. The IR + CGA3 group demonstrated significantly higher SOD-1 mRNA expression compared to the IR group. (e) Reverse transcriptase-PCR (RT-PCR) analysis of inflammatory markers (TLR-4 and MCP-1). The IR + CGA3 group demonstrated significantly lower MCP-1 and TLR-4 mRNA expressions with the IR group. ^*∗*^*p* < 0.05 vs. SO group; ^*∗∗*^*p* < 0.01 vs. SO group; ^*∗∗∗*^*p* < 0.001 vs. SO group. ^#^*p* < 0.05 vs. IR group; ^##^*p* < 0.01 vs. IR group; ^###^*p* < 0.001 vs. IR group.

I/R injury induced myofibroblast expansion as shown by a significantly higher myofibroblast number in the I/R group compared to the SO group (Figures 2(a) and 2(c)). Meanwhile, there were significantly lower myofibroblast numbers in the I/R + CGA2 and I/R + CGA3 groups compared to the I/R group. Myofibroblast expansions might be associated with oxidative stress and antioxidant enzyme reduction. Immunostaining of SOD-1, an antioxidant enzyme, showed positive staining in tubular epithelial cells in the SO group (Figure 2(b)). I/R groups demonstrated reduction of positive signaling compared to SO. This finding was confirmed with RT-PCR of SOD-1 mRNA which showed significantly lower SOD-1 mRNA expression in the I/R group compared to SO. CGA-treated groups had higher SOD-1 mRNA expression; however, only the I/R + CGA3 group had significantly higher expression compared to the I/R group. Immunostaining of SOD-1 also demonstrated positive staining in tubular epithelial cells of the I/R + CGA3 group. Quantification of TLR4 and MCP-1 mRNA expressions confirmed significantly higher numbers in the I/R group compared to SO. RT-PCR revealed only the I/R + CGA3 group had significantly lower TLR-4 and MCP-1 mRNA expressions compared to the I/R group.

### 3.3. CGA Treatment Induced Lower Inflammation and Myofibroblast Expansion

**Figure 3:**
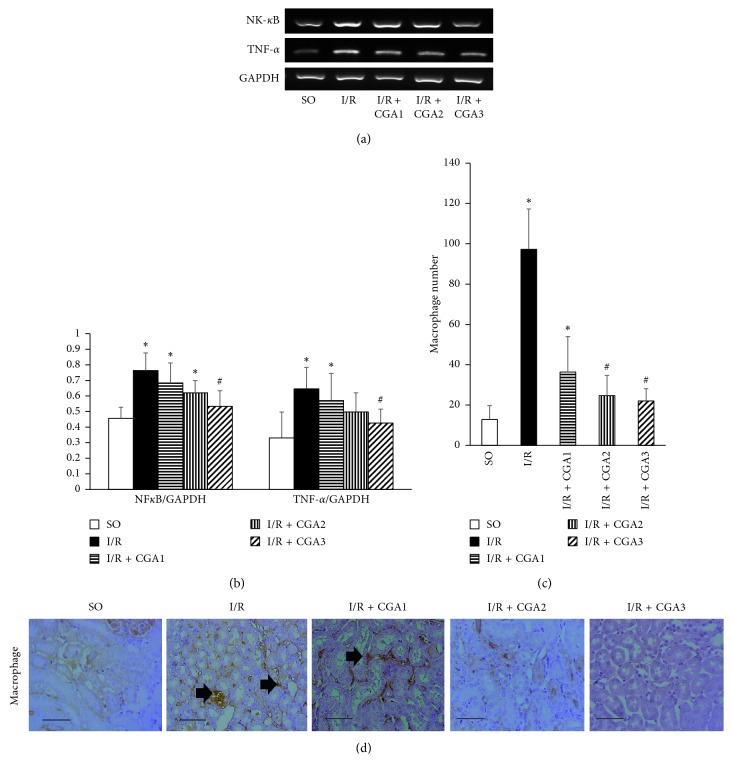
(a, b) Reverse transcriptase-PCR (RT-PCR) analysis of NF-*κ*B and TNF-*α* mRNA expressions. The IR + CGA3 group demonstrated significantly lower NF-*κ*B and TNF-*α* mRNA expressions compared to the IR group. (b) Representative pictures of CD68 immunostaining for macrophage marker. Positive staining is shown in interstitial areas (black arrow). (c) Quantification of the macrophage number showed reduction of the numbers in CGA-treated groups, especially the IR + CGA3 group. (d) Representative pictures of CD68 immunostaining for demonstrating macrophage infiltration (black arrows). ^*∗*^*p* < 0.05 vs. SO group. ^#^*p* < 0.05 vs. IR group.

RT-PCR also revealed increment of inflammation mediators in the IR group. We confirmed higher mRNA expression of NF-*κ*B, a transcription factor, and TNF-*α*, an inflammatory mediator. On the other hand, CGA-treated groups demonstrated lower mRNA expression of NF-*κ*B and TNF-*α*; however, only the I/R + CGA3 group revealed significantly higher mRNA expression compared to the IR group (Figures 3(a) and 3(b)). IHC staining confirmed higher macrophage infiltration after kidney I/R injury as shown by the higher macrophage number in the I/R group compared to the SO group. Lower inflammatory mediator expression in CGA groups associated with the lower macrophage number compared to the I/R group. We found dose-dependent effect of CGA treatment in lowering the macrophage number and inflammation. CGA treatment with the highest dose had the lowest macrophage number and inflammatory mediator expression.

## 4. Discussion

This study highlighted the role of CGA in attenuating kidney I/R injury progression through attenuating tubular injury, inflammation, and myofibroblast formation. Kidney I/R injury leads to AKI and induces tubular injury. Regeneration of the kidneys after injury is characterized by dedifferentiation, proliferation, and migration of survived tubular epithelial cells. These cells replace the dead cells [8]. This condition changes the phenotype of the epithelial cells, from cuboid or columnar shape to squamous-like mesenchymal cells. This process is known as *epithelial-mesenchymal transition* (EMT) [26, 27]. EMT is characterized by the reduction of epithelial marker expression, such as E-cadherin, and upregulation of mesenchymal marker expression, such as vimentin [26]. It seems that regeneration after I/R injury may induce EMT. In this study, we revealed an improvement of tubular injury in the CGA group associated with not only increased tubular proliferation but also reduction of EMT, as shown by the reduction of vimentin and TGF-*β*1 mRNA expressions. Vimentin is known as a key mesenchymal marker, but it also represents the differentiation process. Higher expression of vimentin after ischemia might indicate the ongoing regeneration process [27]. However, it is known that AKI with kidney I/R injury might lead to CKDs. EMT type II after kidney I/R injury also plays a role in renal fibrogenesis with incomplete regeneration and leads to interstitial fibrosis [9]. In our immunohistochemical staining, it appeared that CGA treatment might ameliorate tubular injury, inducing renal epithelial cell proliferation; however, it preserved those epithelial cells while inhibiting the EMT process that leads to fibrosis.

Kidney I/R injury also induces inflammation and ROS production which then activates TGF-*β*1 directly or indirectly [28, 29], causing complementary activation and endothelial injury [5, 30]. Transforming growth factor-*β*1 (TGF-*β*1) may promote EMT and fibrosis [31, 32]. ROS itself has been known to have different actions in epithelial and interstitial cells. ROS induces apoptosis of epithelial cells, while promoting proliferation of interstitial cells [33]. This may cause an increased number of myofibroblasts in the interstitial area after kidney I/R injury.

Our study confirmed that CGA might not only induce proliferation and regeneration of epithelial cells but also may reduce the expansion of myofibroblasts. These findings are associated with an elevation of antioxidant enzyme, SOD-1. The CGA-treated groups revealed upregulation of SOD-1 mRNA expression. This result suggested that the antioxidant effects of CGA may be beneficial in inducing epithelial cell proliferation and inhibiting interstitial cells proliferation. CGA has been known as having antioxidant effects through eliminating free radicals. It has an R-OH sequence that binds to free radicals [19]. ROS itself induces TGF-*β*1 activation in the interstitial area [29].

Our study also demonstrated upregulation of TGF-*β*1 mRNA expression and increase in the myofibroblast number after I/R injury. Increased myofibroblast formation in kidney I/R injury may be associated with TGF-*β*1 upregulation as a main regulator of fibrosis and the EMT process [31, 34]. TGF-*β*1 upregulation may occur temporarily in AKI for regeneration induction, while the upregulation if prolonged can induce CKD and kidney fibrosis [35]. These conditions produce inflammation, fibroblast expansion, extracellular matrix deposition, and fibrosis as final pathways [5]. TGF-*β*1 upregulation occurs significantly 3 hours after injury and continues to 14 days after injury [36, 37]. Our results demonstrated significantly lower TGF-*β*1 mRNA expression in the I/R + CGA3 group compared to the I/R group. This finding demonstrated CGA decreased TGF*β*1 mRNA expression in a dose-dependent manner. CGA treatment has been reported ameliorating liver fibrosis in the carbon tetrachloride- (CCl_4_-) induced hepatic fibrosis model. The reduction of fibrosis was associated with reduction of TGF-*β*1 and *α*-SMA protein level expressions [22]. Similar results were also shown by one study that used CGA treatment for attenuating liver fibrosis in common bile duct ligation. Reduction of fibrosis was also associated with reduction of collagen I and collagen III, TGF-*β*1, and *α*-SMA expressions [8]. Furthermore, CGA also downregulated TGF-*β*1 expression in a subtotal nephrectomy model [38].

Hypoxia after I/R injury leads to upregulation of the NF-*κ*B transcription factor [39] which then induces TGF-*β* upregulation [40], inflammation cytokines, such as TNF-*α*, IL-1*β*, and IL-6 [41], CCL and CXCL subfamily [42], and iNOS [30]. TGF-*β* production can be stimulated by inflammation cytokines in tubular epithelial cells [43]. Focusing on inflammation responses after kidney I/R injury, we investigated TLR-4 signaling and its downstream pathways. Kidney I/R injury induced upregulation of TLR-4 and its downstream signaling and NF-*κ*B, TNF-*α*, and MCP-1 mRNA expressions (Figure 2), which are associated with macrophage infiltration as shown by the higher macrophage number in the I/R group. Meanwhile, CGA treatment attenuated inflammation through reducing TLR-4 signaling (NF-*κ*B, TNF-*α*, and MCP-1) and the macrophage number.

Dead cells release damage-associated molecular patterns (DAMPs) such as heat shock protein HMGB1 which binds to TLR-4 and inducing inflammatory cytokines such as TNF-*α* and IL-1*β* [44]. Increased chemokine from epithelial cells, monocyte and macrophage, and dendritic cells induces inflammatory cells migration such as macrophage, neutrophil, and lymphocyte via CCR1 receptor interaction [42]. ROS and adhesion molecules such as ICAM-1 and *P-selectin* from endothelial cells also increase leucocyte and neutrophil infiltration [30]. Inflammatory cells produce inflammatory chemokines, TNF-*α*, IL-1*β*, and IL-6 [4, 45], and ROS [46], which then exacerbate TGF-*β* production.

TLR-4 increases at 24 hours in endothelial cells and then at 24 hours from epithelial cells. It is sustained for 3–5 days, mainly from tubular epithelial cells [47]. CGA is a phytochemical substance with many functions, such as anti-inflammation [48, 49], antioxidant, and antiapoptosis effects [48]. CGA reduces TNF-*α* protein level expression and mRNA expression of iNOS and COX-2. CGA inhibits release of high mobility group box 1 (HMGB-1) to extracellular, thus inhibiting TLR-4 expression upregulation and NF-*κ*B nuclear translocation [50]. CGA also impedes TLR-4 expression through inhibiting I*κ*B phosphorylation which is associated with NF-*κ*B activation [51]. Anti-inflammatory effect of CGA might be in a dose-dependent manner as shown by significantly lower TLR-4 and MCP-1 mRNA expressions in the CGA + I/R3 group, but not in other groups. Based on the conversion dose method, our dose was similar to the study by Yun et al. (2012); however, they used the liver I/R injury model [23]. TLR-4 signaling activation induces NF-*κ*B activation and then produces inflammatory mediators [4], especially MCP-1 [52]. MCP-1 promotor has two binding sites for NF-*κ*B, which increases in the first 30 minutes after kidney I/R injury [53]. Many cells produce MCP-1, such as neutrophils, endothelial cells, and macrophages [54]. Based on the results of our study, we assumed that reduction of MCP-1 expression might be due to reduction of TLR-4 expression and activation. Although there are many pathways which induce MCP-1 expression after I/R injury, activation of JNK may be associated with MCP-1 expression in I/R injury. Pharmacologically, JNK blockade reduced MCP-1 expression [55]. Tubular epithelial cells also express CD40 ligands that interact with Cd154, which activate MAPK and produce MCP-1 and Il-8 in tubular epithelial cells. This process signals and triggers p38 and ERK1/2 of MAPK [56]. Exploring those signaling pathways in renoprotective effects of CGA on kidney I/R injury may provide better understanding of the beneficial underlying mechanism.

MCP-1 is the main attractant for macrophage recruitment in I/R injury [4]. Types of macrophage depend on the time aspect after I/R injury. During the first 3 days of the injury, M1 macrophage will be activated and then M2 macrophage is activated on day 7 after I/R injury. M1 macrophage releases proinflammatory cytokines due to ROS. Meanwhile, M2 macrophage may play an important role in repair process [5]. Here, we reported that CGA treatment-reduced macrophage infiltration with a significantly lower macrophage number in the I/R + CGA2 and I/R + CGA3 groups compared to the I/R group (Figure 2). The SO group demonstrated positive staining of macrophages which represented resident macrophages in the kidneys. Lower macrophage numbers in CGA-treated groups might be associated with anti-inflammation effects of CGA. Recently, research showed the CGA-attenuated MCP-1 and CD68 expressions in the hepatic steatosis model in mice [57].

## 5. Conclusion

Here, we demonstrated renoprotective effects of CGA on kidney I/R injury through reducing inflammatory mediators and macrophage infiltration, while also reducing EMT and myofibroblast formation.

## Data Availability

The data and materials supporting the conclusion of this research article are included within the article.
